# RKIP Suppresses Breast Cancer Metastasis to the Bone by Regulating Stroma-Associated Genes

**DOI:** 10.1155/2012/124704

**Published:** 2012-02-12

**Authors:** Elena Bevilacqua, Casey A. Frankenberger, Marsha Rich Rosner

**Affiliations:** Ben May Department for Cancer Research, Gordon Center for Integrative Science, The University of Chicago, W421C, 929 East 57th Street, Chicago, IL 60637, USA

## Abstract

In the past decade cancer research has recognized the importance of tumorstroma interactions for the progression of primary tumors to an aggressive and invasive phenotype and for colonization of new organs in the context of metastasis. The dialogue between tumor cells and the surrounding stroma is a complex and dynamic phenomenon, as many cell types and soluble factors are involved. While the function of many of the players involved in this cross talk have been studied, the regulatory mechanisms and signaling pathways that control their expression haven't been investigated in depth. By using a novel, interdisciplinary approach applied to the mechanism of action of the metastasis suppressor, Raf kinase inhibitory protein (RKIP), we identified a signaling pathway that suppresses invasion and metastasis through regulation of stroma-associated genes. Conceptually, the approach we developed uses a master regulator and expression arrays from breast cancer patients to formulate hypotheses based on clinical data. Experimental validation is followed by further bioinformatic analysis to establish the clinical significance of discoveries. Using RKIP as an example we show here that this multi-step approach can be used to identify gene regulatory mechanisms that affect tumor-stroma interactions that in turn influence metastasis to the bone or other organs.

## 1. Introduction

Under normal physiological conditions, the stromal compartment of epithelial tissue regulates homeostasis by maintaining the proper architecture and nutrient levels required for epithelial function. It also serves as an important barrier to cell transformation. However in response to lesions (i.e., wounding) the stromal compartment undergoes changes including the recruitment and activation of fibroblasts, immune, and endothelial cells that in turn provide growth, and matrix remodeling factors, as well as a new blood supply. Similar changes in the stromal compartment have been shown to occur during tumor growth and the importance of the stromal compartment, called “the tumor microenvironment,” in modulating and driving cancer progression has become increasingly evident [1]. The tumor microenvironment has become the subject of intense therapeutic and prognostic interest as its phenotypic and molecular characteristics have been correlated with disease-free survival in multiple tumor types [2].

It is believed that during the first phase of carcinogenesis the tumor microenvironment initially reacts to suppress malignant transformation by maintaining tissue architecture and differentiation. As cancer progresses, however, the local stromal compartment shifts to an activated, growth-promoting state, in many ways similar to an inflammatory state, which is initiated and maintained by continuous paracrine communication between stromal and tumor cells. Stromal components engage in a dynamic signaling circuit with primary tumor cells and coevolve with tumor cells to promote tumor progression to an invasive phenotype [3]. Various stromal components, including vascular cells, pericytes, fibroblasts, inflammatory cells, and extracellular matrix components participate in this cycle [4, 5]. A large number of activated myofibroblasts, characterized by the expression of *α*-smooth muscle actin (*α*-SMA), are frequently found in the stroma of human breast carcinoma and are referred to as carcinoma-associated fibroblasts (CAFs). The precise cellular origin of these activated myofibroblasts is not clear but it has been shown that when inoculated with carcinoma cells, CAFs can promote tumor growth in mouse xenograft models [6]. CAFs secrete high levels of stromal cell-derived factor-1 (SDF-1 or CXCL12), a chemokine that can activate its cognate receptor, CXCR4, which is expressed by many carcinoma cells, and stimulate their proliferation. On the other hand SDF-1 can mediate recruitment of endothelial progenitor cells thus promoting angiogenesis, and it has also been implicated in an autocrine signaling loop that promotes differentiation of normal stromal fibroblasts into myofibroblasts [7].

A number of other cytokines, chemokines, and growth factors secreted by cancer cells themselves or by tumor-associated stromal cells have been shown to sustain tumor cell proliferation and progression through different mechanisms. The list of these autocrine/paracrine factors is constantly growing and includes vascular endothelial growth factor (VEGF), fibroblast growth factor (FGF), transforming growth factor-*β* (TGF-*β*), hepatocyte growth factor (HGF), interleukin-6 (IL-6), and osteopontin (OPN) [8]. An important component of this signaling loop is the recruitment and activation of bone marrow-derived myeloid cells (BMDCs), including macrophages, monocytes, mast cells, and neutrophilis. BMDCs have been shown to play a major role in the development and growth of the primary tumor and also in the subsequent hematological dissemination [9]. BMDCs can in fact contribute to the induction of angiogenesis by activating endothelial cells and are recognized as major determinants of tumor invasion. Secretion of different classes of proteases (matrix metalloproteinases, cathepsins, and serine proteases), produced by stromal and/or tumor cells, has been shown to facilitate cancer cell migration by disrupting cell-cell junctions and promoting invasion of the surrounding tissues by proteolytic degradation of the extracellular matrix (ECM) and the basement membrane.

Metastasis is the primary cause of mortality in breast cancer patients and can emerge many years after the removal of the primary tumor. Metastastic progression is a complicated multistep process which includes at least three discrete stages: (1) epithelial-mesenchymal transition (EMT) leading to migration, invasion, and intravasation; (2) circulation, transportation, and extravasation of cells, which then undergo mesenchymal-epithelial transition (MET); (3) colonization of tumor cells within distal tissues including bone and lung [10]. The efficiency of each of these steps on the way to metastasis is highly affected by interactions with a distinct local microenvironment. Cancer cells interact with an activated stroma during the initial phases of invasion and intravasation, with the bloodstream during hematological dissemination, and finally with the metastatic sites during extravasation and colonization. It is generally believed that each of these stages is highly inefficient, and, in particular, only a very small percentage of the tumor cells that enter the circulatory system are able to colonize and form a tumor at distal sites. This concept highlights the fact that healthy tissues exert a protective function toward invading cancer cells and ensure that order is preserved within the tissue through homeostatic mechanisms. Cancer cells that escape this protective function and are able to modify the surrounding stroma to their own advantage are the ones that will eventually succeed in colonizing new organs.

Many studies have highlighted the concept of tissue tropism: although the blood flow pattern certainly contributes to preferred metastatic sites of specific carcinomas, the complex molecular mechanism of homing metastatic cells is also determined by interactions with the microenvironment at target organs. A number of molecular mediators of this interaction have been revealed by recent publications, and gene expression profiling studies have generated distinct gene expression signatures for organ-specific metastatic variants [10–13]. A major role in the tropism of metastatic cells to different organs is exerted by chemokines and their cognate receptors [14]. Local expression in target tissues is believed to guide metastatic cells to specific destinations as a result of local chemotaxis in combination with induction of invasive properties. As a homing mechanism, metastatic breast cancer cells specifically express functional CXCR4 and CCR7 receptors that induce actin polymerization, formation of pseudopodia, and chemotaxis for directional migration [14]. Interestingly, their respective ligands SDF-1 and CCL21 are mainly distributed in organs that represent the main site of breast cancer metastasis, in particular bone.

Breast cancers metastasize to lung, liver, bone, and brain. Bone metastasis is very common among late-stage breast cancer patients but current treatment methods for bone metastasis are mainly palliative, and more effective disease-modifying therapies are needed. Breast cancer frequently generates osteolytic bone metastasis by secreting a series of growth factors that influence bone matrix and bone stromal cells, tipping the balance to osteolytic bone destruction. In this context tumor-derived factors include angiogenic factors (FGF and VEGF), mediators of immune cell recruitment and activation (TGF*β* and TNF*α*), and mediators of fibroblasts activation (FGF and TGF*β*). Moreover cancer cells promote bone degradation by direct secretion of metalloproteinases (such as MMP1) and collagenase I or through indirect mechanisms by activating osteoclasts. Other tumor-derived cytokines and cell surface/ECM proteins like bone morphogenetic protein (BMP), interleukin-11 (IL-11), osteopontin (OPN), and endothelin-1 participate and feed this vicious cycle. In this scenario bone reabsorption by osteoclasts releases a number of growth factors embedded in the bone matrix including insulin-like growth factors (IGFs), TGF-*β*, platelet-derived growth factor (PDGF), and BMP which become part of this signaling circuit that push osteolytic lesions.

Gene expression profiling of a bone-tropic subpopulation of the breast cancer cell line MDA-MB-231 has revealed a “bone metastasis signature” (BMS) [11]. As expected, the most highly overexpressed genes in the BMS encode mostly cell surface and secreted proteins that alter the bone microenvironment in order to facilitate growth of metastases and formation of osteolytic bone lesions as described above. The BMS includes OPN, connective tissue growth factor (CTGF), fibroblast growth factor 5 (FGF5), the osteoclast-activating cytokine IL-11, CXCR4, and MMP1 as well as many other genes. Expression of these genes in the primary tumor has multiple functions including: (i) targeting cells specifically to the bone microenvironment via homing factor CXCR4; (ii) facilitating colonization of the bone via expression of bone extracellular matrix degrading enzymes (MMP1, ADAMTS1); (iii) activating osteoclasts and favoring adhesion to the bone surface through OPN [15]. Overexpression of individual genes in the signature led to only a marginal increase in bone metastasis, whereas coexpression of multiple genes dramatically increases both the rate and incidence of bone metastasis [11]. This concept implies that these genes cooperate to push the metastatic phenotype and may not be highly effective if isolated from their signaling context. This observation also highlights the importance of understanding the master molecular mechanisms that regulate expression of genes in order to develop target therapies that affect their combined expression rather than an isolated component.

## 2. RKIP Defines Ways to Suppress Invasion and Metastasis

To understand the mechanisms by which metastasis is regulated, we have focused on identifying key signaling pathways that can inhibit breast cancer metastasis to the bone. Metastasis suppressors define a class of proteins that do not affect primary tumor growth but instead regulate one or more steps in the process leading to metastasis: invasion, intravasation, circulation, extravasation, and colonization of the secondary site [16]. Raf kinase inhibitory protein (RKIP) was initially shown to function as a metastasis suppressor in a prostate xenograft mouse model [17]. More recently, we have shown that RKIP also suppresses metastatic progression to bone in breast tumor xenografts [18]. Furthermore, we demonstrated that RKIP inhibits breast cancer invasion, intravasation, and bone metastasis via a signaling pathway involving induction of the microRNA let-7. Specifically, inhibition of the Raf/MEK/MAP kinase cascade by RKIP leads to inhibition of Myc activation. Myc is a transcriptional activator of LIN28, which in turn inhibits let-7 maturation. Consistent with the role of this signaling cascade, LIN28 has been implicated in breast cancer progression and let-7 functions as an inhibitor of breast tumor formation [19]. We also showed that let-7 inhibits invasion in part via suppression of the chromatin remodeling factor high mobility group AT-hook 2 (HMGA2). HMGA2 in turn activates Snail, a transcription factor that promotes the epithelial-mesenchymal transition (EMT), a process that favors the acquisition of an invasive phenotype. To understand how this upstream signaling cascade regulates genes that are involved in the crosstalk with the tumor microenvironment, thus affecting breast cancer metastasis to the bone, we sought to identify relevant metastatic genes that function downstream of the RKIP/let-7 axis.

As a means of identifying signaling pathways downstream of a key metastasis regulator in cancer, the Rosner and Minn groups developed a novel interdisciplinary approach that utilizes clinical data from breast tumors to generate and test hypotheses [20]. The basic idea is to determine whether a discrete set of genes are targets of inhibition by a metastasis suppressor, in this case RKIP. If RKIP inhibits expression of these genes, then their expression levels in breast tumors should inversely correlate with RKIP expression. Once we identified genes that inversely correlate with RKIP in patients' tumors, we tested them experimentally *in vitro* using breast tumor cell lines and *in vivo* using a xenograft mouse model. Finally, having determined which genes regulate metastasis in experimental breast tumor models, we validated their clinical significance by further bioinfomatic analysis using independent breast tumor data.

Using this approach, we identified a number of RKIP-regulated let-7 targets including HMGA2 and a novel target, BTB-and-CNC homology 1 (BACH1). A leucine zipper transcription factor, BACH1, has been linked previously to senescence and heme oxidation but has never been correlated to cancer progression [21]. Experimental validation using a xenograft mouse model confirmed that RKIP and let-7 suppress BACH1 and HMGA2 expression and showed that BACH1 promotes invasion, intravasation, and bone metastasis of breast cancer cells.

To test the hypothesis that RKIP is a potential regulator of genes implicated in the development of bone metastasis, we performed a similar bioinformatic analysis. We initially determined whether RKIP expression inversely correlates to the expression of bone metastasis signature (BMS) genes [11]. We assembled several cohorts of primary breast tumor expression array data and performed gene set analysis (GSA) correlating the expression levels of the set of BMS genes to RKIP expression. As expected, we found a negative correlation between RKIP and BMS genes in two independent gene expression data sets of 443 and 871 breast cancer patients [20]. Thus, when RKIP is expressed, BMS genes show low expression levels and vice versa. Having found a significant correlation, we experimentally tested five genes that were previously implicated as promoting breast tumor bone metastasis by regulating interactions of cancer cells with the stroma. Of these, we were able to demonstrate that RKIP inhibits expression of MMP-1, CXCR4, and OPN thus affecting the ability of metastatic cells to create an osteolytic bone environment via crosstalk with stromal cellular and noncellular components.

Finally, we determined experimentally the relationship between RKIP, let-7, the two let-7 targets, HMGA2 and BACH1, and the three BMS genes. Interestingly, knockdown of BACH1 suppressed the BMS genes MMP1 and CXCR4 but not OPN while HMGA2 knockdown suppressed CXCR4, and OPN but not MMP1. Additionally, we could partially reverse the effects of HMGA2 and BACH1 knockdown on invasion and metastasis by overexpressing their target BMS genes MMP1, CXCR4 and OPN. The simultaneous overexpression of the three BMS genes together showed a more profound effect on the metastatic phenotype compared to the overexpression of a single gene. These results suggest that the coordinate regulation of genes with different metastasis-promoting functions is a prerequisite for efficient metastatic spread.

Having determined which genes regulate metastasis in experimental breast tumor models, we defined a signaling pathway signature termed the RKIP pathway metastasis signature (RPMS) that we could use to further validate the clinical significance of our findings [20]. While typical gene expression signatures do not implicate any regulatory relationship between the genes in the signatures, the RPMS is based upon experimentally validated regulatory relationships between the components of the pathway. Bioinfomatic analysis using breast tumor data showed that the complete RPMS can predict greater risk for metastasis in patients. By contrast, the individual genes in the RPMS pathway were unable to predict metastasis-free survival. Taken together, these results highlight the importance of evaluating both regulators of tumor metastasis as well as genes that interact with the cellular signaling environment in order to be able to predict metastatic risk.

## 3. Significance

**Figure 1:**
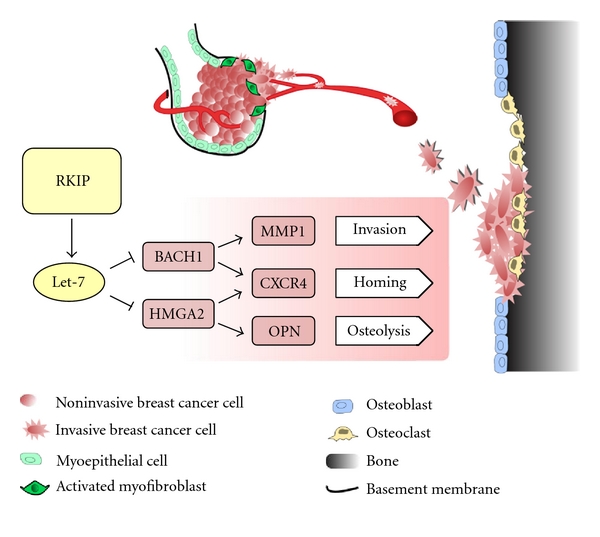
Schematic representation of the RKIP signaling pathway and its effects on metastatic progression to the bone.

The results described here reveal a novel regulatory mechanism, controlled by the RKIP signaling pathway, that modulates the dialogue between breast tumor cells and the microenvironment and affects metastatic progression to the bone (Figure 1). Specifically, recent studies demonstrate that BACH1 and HMGA2 are key targets for inhibition by the RKIP signaling pathway via a let-7-dependent mechanism. Furthermore, BACH1 and HMGA2 promote the development of bone metastasis by inducing expression of genes (MMP1, CXCR4, and OPN) that regulate properties of the stromal compartment at the target organ site. Finally, since OPN is regulated exclusively by HMGA2 and MMP1 by BACH1, the signaling pathways downstream of RKIP exhibit a degree of specificity. While the function of these genes has been studied extensively in the past in the context of metastasis, the regulatory mechanisms and signaling pathways that control their expression were thus far incompletely investigated. The ability to manipulate a set of bone metastasis genes through a common upstream regulator such as RKIP reveals potential therapeutic targets that could have a profound impact on prevention of metastasis in breast cancer patients.
